# Telomere Length in Metaphase Chromosomes of Human Triploid Zygotes

**DOI:** 10.3390/ijms22115579

**Published:** 2021-05-25

**Authors:** Anna A. Pendina, Mikhail I. Krapivin, Olga A. Efimova, Andrei V. Tikhonov, Irina D. Mekina, Evgeniia M. Komarova, Alla S. Koltsova, Alexander M. Gzgzyan, Igor Yu. Kogan, Olga G. Chiryaeva, Vladislav S. Baranov

**Affiliations:** D.O. Ott Research Institute of Obstetrics, Gynecology and Reproductology, Mendeleevskaya Line 3, 199034 Saint Petersburg, Russia; pendina@mail.ru (A.A.P.); krapivin-mihail@mail.ru (M.I.K.); tixonov5790@gmail.com (A.V.T.); irendf@mail.ru (I.D.M.); evgmkomarova@gmail.com (E.M.K.); rosenrot15@yandex.ru (A.S.K.); agzgzyan@gmail.com (A.M.G.); ikogan@mail.ru (I.Y.K.); chiryaeva@mail.ru (O.G.C.); baranov@vb2475.spb.edu (V.S.B.)

**Keywords:** telomere length, human zygote, metaphase chromosomes, maternal and paternal pronuclei, age, sperm quality, parental origin

## Abstract

The human lifespan is strongly influenced by telomere length (TL) which is defined in a zygote—when two highly specialised haploid cells form a new diploid organism. Although TL is a variable parameter, it fluctuates in a limited range. We aimed to establish the determining factors of TL in chromosomes of maternal and paternal origin in human triploid zygotes. Using Q-FISH, we examined TL in the metaphase chromosomes of 28 human triploid zygotes obtained from 22 couples. The chromosomes’ parental origin was identified immunocytochemically through weak DNA methylation and strong hydroxymethylation in the sperm-derived (paternal) chromosomes versus strong DNA methylation and weak hydroxymethylation in the oocyte-derived (maternal) ones. In 24 zygotes, one maternal and two paternal chromosome sets were identified, while the four remaining zygotes contained one paternal and two maternal sets. For each zygote, we compared mean relative TLs between parental chromosomes, identifying a significant difference in favour of the paternal chromosomes, which attests to a certain “imprinting” of these regions. Mean relative TLs in paternal or maternal chromosomes did not correlate with the respective parent’s age. Similarly, no correlation was observed between the mean relative TL and sperm quality parameters: concentration, progressive motility and normal morphology. Based on the comparison of TLs in chromosomes inherited from a single individual’s gametes with those in chromosomes inherited from different individuals’ gametes, we compared intraindividual (intercellular) and interindividual variability, obtaining significance in favour of the latter and thus validating the role of heredity in determining TL in zygotes. A comparison of the interchromatid TL differences across the chromosomes from sets of different parental origin with those from PHA-stimulated lymphocytes showed an absence of a significant difference between the maternal and paternal sets but a significant excess over the lymphocytes. Therefore, interchromatid TL differences are more pronounced in zygotes than in lymphocytes. To summarise, TL in human zygotes is determined both by heredity and parental origin; the input of other factors is possible within the individual’s reaction norm.

## 1. Introduction

Telomeres are repetitive DNA sequences capping the ends of linear chromosomes and featuring a specific structure. Telomeres consist of a varying number of tandemly repeated hexanucleotides, shelterin proteins and telomere repeat-containing RNAs [1,2,3,4,5]. Telomeres distinguish chromosome ends from double-stranded breaks and ensure genome stability [6,7,8,9]. Critical telomere shortening triggers adverse events, such as cell cycle arrest, apoptosis, an altered, pro-inflammatory secretory profile or chromothripsis [10,11,12]. 

Telomeres are among the most dynamic genome structures. Telomere length (TL) remains in continuous dynamic equilibrium during ontogenesis and the transfer of genetic information from one generation to another. On the one hand, telomeres inevitably shorten during cell divisions [13,14,15,16] and as a result of exposure to external detrimental factors such as reactive oxygen species [17,18]. On the other hand, they can lengthen due to telomerase reverse transcriptase activity [19] and through a recombination-based mechanism termed alternative lengthening of telomeres (ALT) [20,21].

TL has been extensively investigated in human somatic cells [15,22,23,24,25]. By contrast, TL in human germ cells is the least explored area, even though it is an issue of paramount importance, considering the role of TL in reproductive disorders [26,27,28,29]. Studies of TL in oocytes are sparse and primarily examine cells obtained during assisted reproduction [27,30,31]. The available research suggests that telomeres in human oocytes are shorter than those in somatic cells [32,33] and shorten progressively with a woman’s age [29,34,35], while women’s somatic cells feature longer telomeres than men’s [36,37,38,39]. The opposite is true regarding TLs in male cells: while TL in sperm is among the highest [40,41] and increases with age [30,42,43,44,45,46], men’s somatic cells feature shorter telomeres than women’s [36,37,38,39]. Therefore, humans are a fascinating case of sexual dimorphism in TL, both in somatic and germ cells. Understanding how this sexual dimorphism manifests during the initial stage in a new organism’s development after the genetic materials from a sperm and an oocyte combine in a zygote is of great interest. To that end, the present study aims to examine TLs in human triploid zygotes for chromosomes inherited from sperm and oocytes and to analyse their relationship with parental age and sperm quality parameters.

## 2. Results

### 2.1. Patients and Samples

We examined TL in 28 triploid zygotes obtained from 22 couples. For ethical reasons, diploid zygotes could not be included in our study. However, there is some evidence suggesting that critical developmental events in triploid human embryos are similar or identical to those in normal ones. Triploid human embryos are capable of implantation and even a full-term development [47]. Kattera and Chen reported a case of microsurgical enucleation of the extrapaternal pronucleus from a tripronucleate human zygote followed by embryo transfer, pregnancy and a normal birth [48]. Thus, it is possible that inheritance and reprogramming of TL in triploid zygotes may, at least in part, reflect those in diploid ones. 

The participants’ demographic information and semen parameters, and the parental origin of pronuclei in the zygotes are presented in Table 1.

### 2.2. Pronuclei Synchronisation at the Metaphase Stage

**Figure 1:**
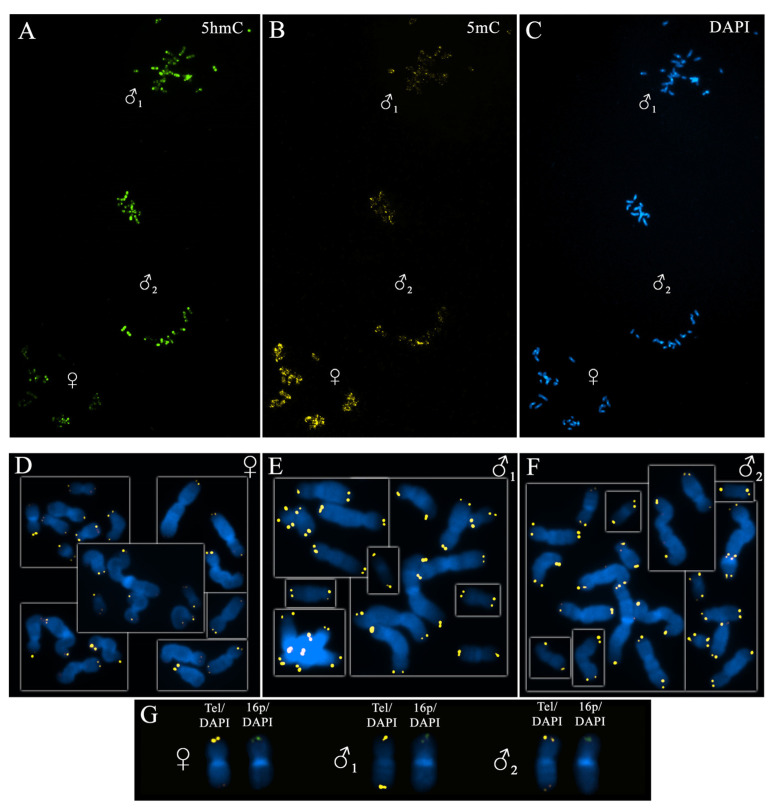
Parental origin identification and telomere detection in the metaphase chromosomes of a human triploid zygote. The chromosomes’ parental origin was determined through immunocytochemical staining with anti-5-hydroxymethylcytosine (5hmC) (**A**) and anti-5-methylcytosine (5mC) (**B**) antibodies and DAPI counterstaining (**C**) (magnification 10 × 20, var. × 0.63 for **A**–**C**). Chromosomes from pronuclei of different parental origin show contrasting 5mC and 5hmC patterns: those from a maternal pronucleus are hypermethylated and hypohydroxymethylated, while those from the paternal pronuclei are hypomethylated and hyperhydroxymethylated. Telomeres in chromosomes from maternal (**D**) and paternal (**E**,**F**) pronuclei were detected through fluorescent in situ hybridisation (FISH) with telomeric DNA probes, and the chromosomes were stained with DAPI (magnification 10 × 100 for **D**–**F**). Panel **G** shows three homologues of chromosome 16 from a maternal and two paternal pronuclei after FISH with telomeric (Tel/DAPI) and subtelomeric 16p (16p/DAPI) DNA probes.

After fertilisation, the male and the female pronuclei are not formed in the zygote simultaneously. Pronucleus formation normally occurs within several hours after the second polar body extrusion but may vary significantly in different oocytes. After we registered three pronuclei in a zygote, and therefore once its further culturing was no longer practical, we added colchicine to the culture medium to achieve the synchronisation necessary for an objective comparative assessment of TL in pronuclei of different parental origin. This approach enabled us to evaluate the TLs of oocyte- and sperm-derived chromosomes in zygotes at the same cell cycle stage—the metaphase of mitosis. We analysed metaphase chromosomes from 84 parental pronuclei across 28 zygotes. In some of the zygotes, parental chromosomes were visible as separate haploid sets (Figure 1); in others, the maternal and paternal chromosomes were mixed and were present as a single metaphase plate. For cytogenetic analysis, we applied the chromosome banding technique with DAPI.

### 2.3. Determining the Parental Origin of Chromosomes

The parental origin of chromosomes was determined immunocytochemically with the use of specific anti-5-methylcytosine (5mC) and anti-5-hydroxymethylcytosine (5hmC) antibodies [30,49,50]. This technique ensured the reliable identification of sperm-derived (paternal) chromosomes through weak DNA methylation and strong hydroxymethylation and oocyte-derived (maternal) chromosomes through strong DNA methylation and weak hydroxymethylation (Figure 1). In 24 out of 28 triploid zygotes, we identified two chromosome sets inherited from the sperm and one inherited from the oocyte, while four other zygotes contained one paternal and two maternal sets (Table 1).

### 2.4. Telomere Length Analysis

TL in the metaphase chromosomes was assessed through hybridisation signal fluorescence intensity after quantitative fluorescence in situ hybridisation (Q-FISH), which is the optimal technique for single-cell analysis. In view of the fact that chromosomes inherited from the sperm undergo protamine replacement with histones in the zygote, their condensation level is different from that of chromosomes inherited from the oocyte. To account for the gap, we calculated relative TLs instead of absolute values. Relative TLs were calculated by dividing telomere fluorescence by the fluorescence of the reference hybridisation signal. As a reference, we used the subtelomeric region of the short arm of chromosome 16 (16p), which is characterised by exceptionally low, verging on negligible, interindividual variability. To evaluate the possible influence of the chromosome’s parental origin on the 16p subtelomere’s fluorescence intensity, we compared this parameter for paternal and maternal chromosomes using the Wilcoxon test, which did not show a significant difference (*p* = 0.28).

To ensure objective results, we measured relative TLs in the same chromosome for every pronucleus across all of the 28 analysed zygotes. We selected chromosome 16 for analysis because it can be reliably identified due to its pronounced centromeric heterochromatin and reference subtelomeric region, marked by the FISH signal. 

To justify this approach, we carried out correlation analysis and checked the strength and direction of correlation between the TL in chromosome 16 and TLs in other chromosomes of the same chromosome set. For this analysis, the intensity of telomeric fluorescent signals was measured for each chromosome in a total of 33 parental chromosome sets from 11 zygotes. For each chromosome in a chromosome set, we conducted four measurements: two in the short arms and two in the long arms of the sister chromatids. The arithmetic mean for telomere fluorescence was calculated for each chromosome set by summarising all of the telomere fluorescence values and dividing the sum by the total number of values. For the 16p subtelomere FISH signal intensity (reference), two measurements were conducted on each chromosome set: one on each sister chromatid. The arithmetic mean for 16p subtelomere fluorescence was then calculated. The mean relative telomere fluorescence value for each chromosome set was calculated by dividing its mean telomere fluorescence by the mean 16p subtelomere fluorescence. 

**Figure 2:**
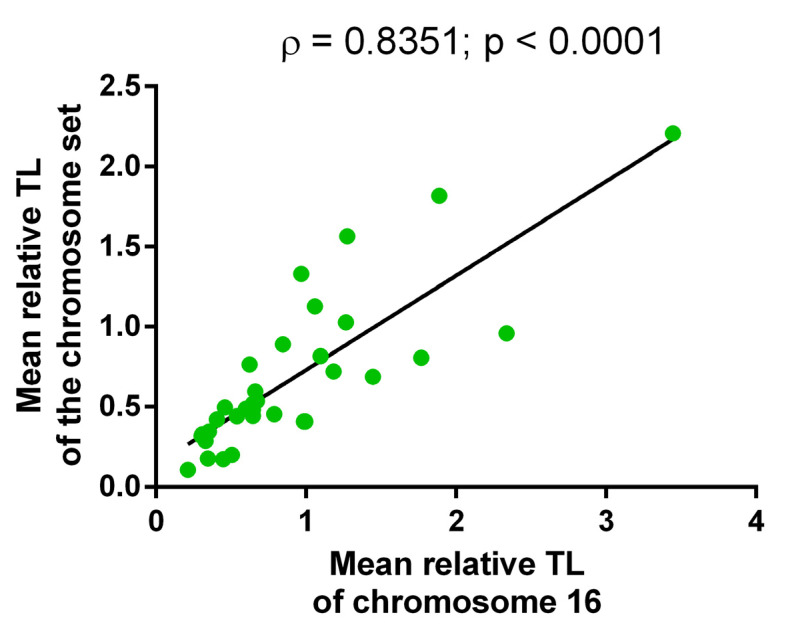
The correlation between the mean relative telomere length (TL) of chromosome 16 and that of other chromosomes in the same metaphase assessed in 33 pronuclei across 11 human triploid zygotes (Spearman test, ρ = 0.8351; *p* < 0.0001).

The Spearman test showed a strong positive correlation between the mean relative TL of chromosome 16 and that of the whole metaphase (ρ = 0.8351, *p* < 0.0001), attesting to the applicability of our approach (Figure 2).

### 2.5. The Difference in Telomere Length between Chromosomes of Different Parental Origin

**Figure 3:**
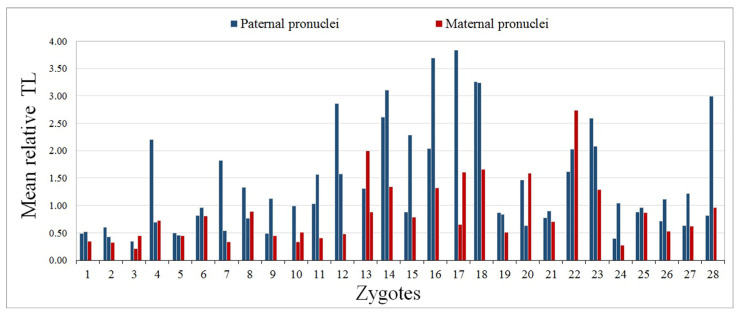
Mean relative telomere lengths (TLs) of chromosome 16 from paternal (blue) and maternal (red) pronuclei of 28 human triploid zygotes. Paternal telomeres are longer than maternal ones (the Wilcoxon test, *p* < 0.0001).

We carried out a comparative analysis of mean relative TLs in the chromosomes of paternal and maternal origin across 28 zygotes. The comparison involved 24 zygotes with one female and two male chromosome sets, and four zygotes with one male and two female sets, totalling 56 comparison pairs (Table 1). In most cases, the mean relative TL in sperm-derived chromosomes exceeded that in oocyte-derived ones, sometimes reaching up to a 5–6-fold difference (Figure 3). The comparison of paired samples with the Wilcoxon test yielded a significant difference (*p* < 0.0001). Therefore, in human triploid zygotes, TL in paternal chromosomes is significantly higher than in maternal ones.

### 2.6. Analysis of Correlations between Telomere Lengths, Age and Semen Parameters

**Figure 4:**
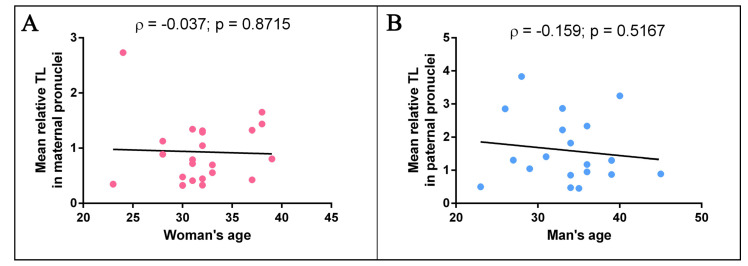
The relationships between telomere length (TL) in metaphase chromosomes from human triploid zygotes and the parents’ age. Scatter plot of the mean relative TL in paternal chromosomes and paternal age: the correlation is not statistically significant (ρ = −0.159, *p* = 0.5167, Spearman test) (**A**). Scatter plot of the mean relative TL in maternal chromosomes and maternal age: the correlation is not statistically significant (ρ = −0.037, *p* = 0.8715, Spearman test) (**B**).

**Figure 5:**
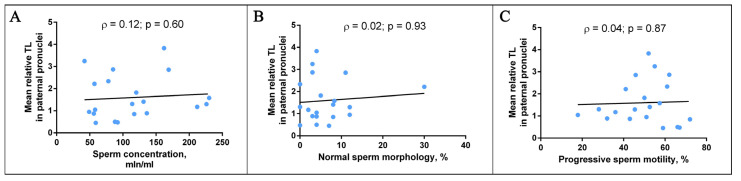
The relationships between telomere length (TL) in paternal chromosomes from human triploid zygotes and sperm parameters. Scatter plots of the mean relative TL in paternal chromosomes and sperm concentration (**A**), normal sperm morphology (**B**) and progressive sperm motility (**C**). In neither case did the Spearman test show a statistically significant correlation.

To investigate the possible relationship between age and TL, we used the Spearman test and carried out a correlation analysis of the two parameters. We did not detect a significant correlation between the mean relative TL in paternal chromosomes and the paternal age (ρ = −0.159, *p* = 0.5167) or between the mean relative TL in maternal chromosomes and the maternal age (ρ = −0.037, *p* = 0.8715) (Figure 4). Moreover, we did not identify a correlation of the mean relative TL in paternal chromosomes with the sperm concentration (ρ = 0.12, *p* = 0.60), normal sperm morphology (ρ = 0.02, *p* = 0.93) or progressive sperm motility (ρ = 0.04, *p* = 0.87) (Figure 5). Therefore, such factors as age and specific sperm parameters—sperm concentration, normal sperm morphology and progressive motility—are not associated with TL in human triploid zygotes.

### 2.7. A Comparison of Intraindividual and Interindividual Telomere Length Variability

To assess the input of heredity, we compared intraindividual (intercellular) and interindividual TL variability.

Intraindividual (intercellular) variability was assessed based on TL comparison across pronuclei inherited from different gametes of the same individual. Based on parental origin analysis, we calculated the number of pronuclei formed from each individual’s gametes in all of the obtained zygotes and treated this number as the number of gametes obtained from each respective individual (Table 2). This number varied from one to six across the participants under study. For the gametes of 27 individuals whose relative TL was measured across two or more pronuclei, we calculated the mean value. Further on, we calculated the fold change between the value measured in each pronucleus and the mean value across all of the pronuclei within the zygote(s) of every individual, for a total of 48 values (Table 2). Using the Mann–Whitney U test, we compared the fold change values calculated for maternal and paternal pronuclei. As no difference was found (*p* = 0.32), we combined the values into one group and determined the indicators of intraindividual (intercellular) variability: a variance of 0.09, a standard deviation of 0.3, a standard error of the mean of 0.04 and a coefficient of variation of 22.66%. 

To assess interindividual variability, we estimated the fold change between the relative TL across all of the pronuclei in the same individual and the mean value for all individuals within the female and the male groups (22 individuals each). Based on the absence of differences between the female and the male groups as per the Mann–Whitney U test (*p* = 0.97), we grouped the values calculated for opposite-sex individuals and determined indicators reflecting interindividual variability: a variance of 0.42, a standard deviation of 0.65, a standard error of the mean of 0.1 and a variation coefficient of 36.8%. 

**Figure 6:**
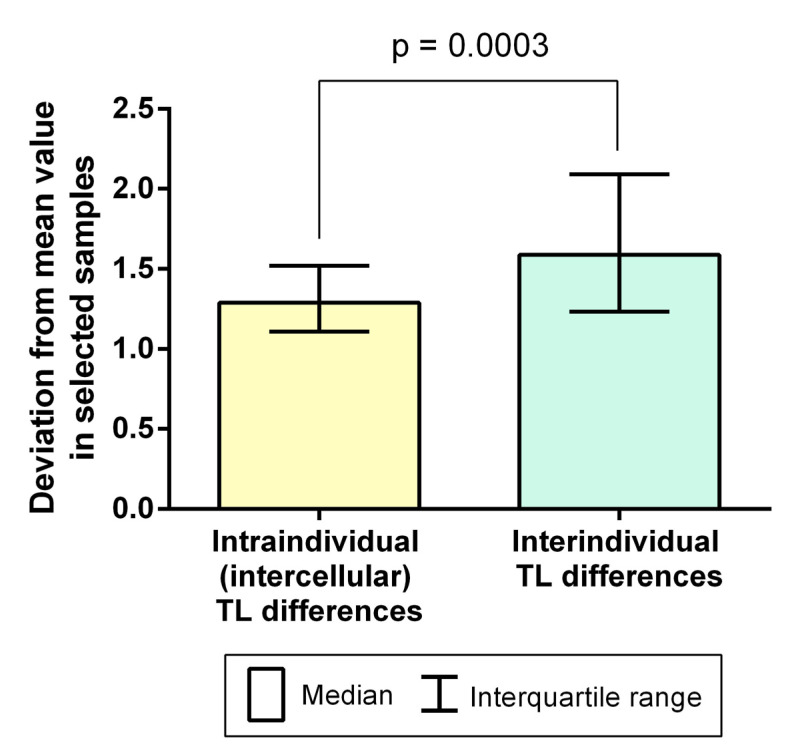
A significant excess of interindividual variability over intraindividual (intercellular) variability calculated based on telomere length (TL) assessment in triploid zygotes (the Mann–Whitney U test, *p* = 0.0003).

We compared fold change values characterising intraindividual (intercellular) and interindividual variability with the non-parametric Mann–Whitney U test, which showed a higher interindividual variability (*p* = 0.0003) (Figure 6). The variance of fold change values reflecting interindividual variability was higher compared to that of fold change values reflecting intraindividual (intercellular) variability (Levene’s test, F = 20.38, *p* < 0.0001). 

Therefore, interindividual variability significantly exceeds intraindividual (intercellular) variability, attesting to the role of heredity in determining TL. 

### 2.8. Interchromatid Telomere Length Differences in Triploid Zygotes and Adult Lymphocytes

We assessed TLs in the zygotes after the completion of the S-phase of the cell cycle and before the first cleavage division, in metaphase chromosomes comprising two chromatids each. To investigate interchromatid differences reflecting changes in the telomere regions of the daughter chromatid compared to the mother chromatid, we calculated TL ratios between sister chromatids. The calculation was carried out for chromosomes 1, 9 and 16, which could be easily identified due to their large heterochromatic regions. To calculate the ratio between sister chromatids, we divided the higher value of the telomere fluorescence intensity by the lower value. Two values were calculated for each of the chromosomes 1, 9 and 16: the ratio between TLs in the sister chromatids in the short chromosome arms and those in the long chromosome arms, except for cases of overlapping chromosomes, in which telomere fluorescence could not be reliably measured. We then grouped the values obtained by parental origin: 274 values in chromosomes from 52 paternal pronuclei and 162 values in chromosomes from 32 maternal ones. As a baseline group, we assessed TLs in the sister chromatids of chromosomes 1, 9 and 16 of PHA-stimulated lymphocytes of 11 adult individuals in 22 metaphases (a total of 209 values). 

**Figure 7:**
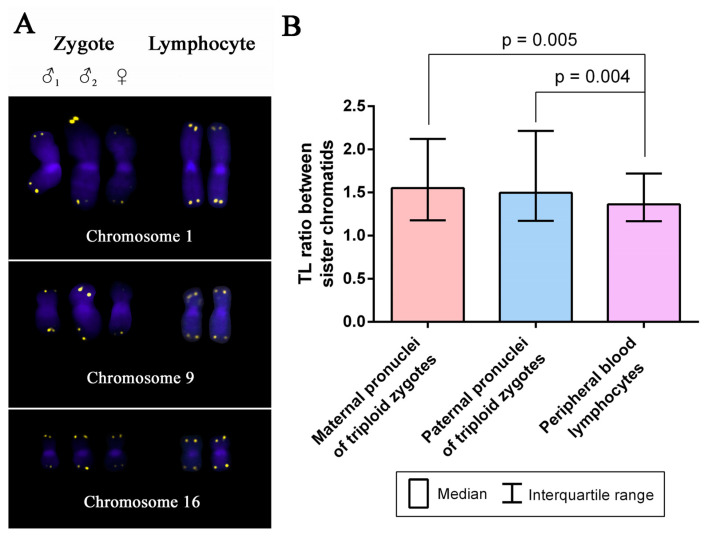
Interchromatid telomere length (TL) difference in metaphase chromosomes of human triploid zygotes and PHA-stimulated lymphocytes. (**A**) Homologous chromosomes 1, 9 and 16 from a triploid zygote with one maternal and two paternal chromosome sets and from a PHA-stimulated adult lymphocyte after fluorescent in situ hybridisation (FISH) with telomeric DNA probes and DAPI-staining. Sister chromatids in zygotic chromosomes show pronounced TL asymmetry. (**B**) Column bar charts of TL ratios between the sister chromatids of chromosomes 1, 9 and 16 within maternal and paternal chromosomes of triploid zygotes and in PHA-stimulated peripheral blood lymphocytes of healthy adult donors. Interchromatid TL difference in maternal and paternal chromosomes in human triploid zygotes is higher than in PHA-stimulated lymphocytes (the Mann–Whitney U test, *p* = 0.005 and *p* = 0.004, respectively).

To estimate interchromatid TL differences, we compared the values obtained in chromosomes from zygote pronuclei of different parental origin with those from PHA-stimulated adult lymphocytes. Interchromatid TL ratios did not differ between chromosomes from maternal and paternal pronuclei (the Mann–Whitney U test, *p* = 0.91). However, they were significantly higher in both maternal and paternal pronuclei compared to adult lymphocytes (*p* = 0.005 and *p* = 0.004, respectively) (Figure 7). Therefore, interchromatid TL differences are more pronounced in zygotes than in PHA-stimulated lymphocytes.

## 3. Discussion

We identified two noteworthy phenomena in our analysis. On the one hand, the low TL variability across the gametes of one individual and the high TL variability across different individuals’ gametes attest to a dominant role of heredity in determining TL in paternal and maternal pronuclei at the zygote stage. Multiple studies on the lymphocytes of monozygotic and dizygotic twins [51,52,53], amniocytes [52] and fibroblasts [52] also attest to the heritability of TLs. This suggestion is further confirmed by the data on TL differences between homologous chromosomes in an individual’s cells maintained throughout the individual’s life [53].

On the other hand, we established that telomeres of paternal chromosomes in the zygote are longer than maternal ones. This premise suggests a certain “imprinting” of TLs. In other words, TL in a zygote is determined not only by the traits of the individual from whom they were inherited but also by the individual’s sex. Therefore, there is a possibility of sex-specific TL changes during spermatogenesis or oogenesis. Most likely, however, these changes occur only within the reaction norm of this trait.

As our study suggests, the impact of a parent’s sex on TL in the zygote is considerably more pronounced than that of a parent’s age. Although the effect of age has been observed in male gametes [30,42,43,44,45], it is less significant than the parental origin, does not introduce such strong variability and cannot be statistically registered against the pronounced impact of this vital factor at the pronucleus stage. In this regard, identifying a significant difference would require a much larger sample set and a wider age range; therefore, the question remains open for now.

In addition, our study did not find evidence of any correlation between TL in the male pronucleus and such parameters of sperm quality as concentration, progressive motility or normal morphology. While some studies registered correlations between TL in spermatozoa and total sperm count [54,55], sperm concentration [56], motility [57,58], sperm DNA fragmentation [57,58] and oligozoospermia [54], other studies did not show such associations [30,55,57,58]. There is a possible explanation for such discrepancies in the results. We may surmise that all individuals, including those from different age groups, possess several germ cell generations but that fertilisation involves only the gametes in which TL fits within a specific range. In other words, a certain selection by TL occurs both in male and female gametes. There is a probability that gametes with critically short or excessively long telomeres cannot participate in fertilisation.

Longer telomeres in the paternal chromosomes of a zygote could be explained by the fact that, during cleavage divisions, in the absence of active telomerase, the zygote needs a quick mechanism preventing telomeres from shortening at the least and elongating at the most. The most likely mechanism is ALT, as telomerase, which is most active at the blastocyst stage [32,33], works slowly [59], in contrast to fast and effective recombination between the homologous regions of telomeric DNA [20,21,60,61,62,63,64]. Most likely, chromosomes inherited from sperm serve as a template for the elongation of chromosomes inherited from the oocyte. Meanwhile, the global demethylation, both active and replication-dependent, which occurs throughout cleavage divisions [49,50,64,65,66], strongly promotes recombination between the homologous regions of telomeric DNA [67,68,69]. Another factor supporting the recombination-based mechanism is the TL difference between sister chromatids, which, as we have demonstrated, is more pronounced in zygotes than in adult lymphocytes. The results of studies on immortalised human cell lines and tumours also confirm the probability of telomere–telomere recombination [70].

Our findings suggest that at least two factors determine TL in human zygotes: heredity and parental origin. The input of other factors is possible within the reaction norm determined by individual traits. Therefore, the length of telomeres in a newly formed organism is, on the one hand, under strict genetic control, which is most likely closely linked to the epigenetic reprogramming of the embryo genome, and, on the other hand, retains a certain degree of variability, which is influenced by internal and external factors—directly or via cell biosensors, such as epigenetic mechanisms [71]. The sexual dimorphism of TLs in genomes of different parental origin may become less pronounced during cleavage divisions, as shorter telomeres in maternal chromosomes are compensated for by longer telomeres in paternal ones. However, to validate this assumption, further detailed investigation of TLs during cleavage divisions of the human embryo is required.

## 4. Materials and Methods

### 4.1. Collection of Human Zygotes

This study included human triploid zygotes that were routinely subjected to negative selection in IVF protocols. Zygotes were produced through conventional IVF at the D.O. Ott Research Institute of Obstetrics, Gynecology and Reproductology (Saint Petersburg, Russia). Shortly after, cumulus–oocyte complexes were retrieved after controlled ovarian hyperstimulation as described earlier [72]. Oocytes were rinsed in Flushing Medium (Origio, Målov, Denmark) prewarmed to +37 °C and were incubated in the ISM1 medium (Origio, Målov, Denmark) at +37 °C in an atmosphere of 5% CO_2_ for 3 h. Routinely prepared sperm was then added for IVF. The presence and the number of pronuclei were examined after 20 h. A total of 28 triploid zygotes from 22 couples were included in the study.

### 4.2. Collection and Culturing of Human Lymphocytes

The peripheral blood lymphocytes were donated for the study by 11 healthy karyotypically normal volunteers aged 18–54 years. The lymphocytes were cultured for 72 h at +37 °C in the presence of phytohemagglutinin (PHA) (PanEco, Moscow, Russia) according to the standard protocol with minor modifications used in our laboratory [73].

### 4.3. Chromosome Preparation

For chromosome preparations from the tripronucleate zygotes, 5 µL of 0.1% colchicine (Merck) was added to the culture medium at the point of visualisation of three pronuclei. After the pronuclei had dissolved (i.e., in 6–12 h), the zygotes were fixed on glass slides as described previously [74], with minor modifications [65,73]. 

Preparations of metaphase chromosomes from the PHA-stimulated lymphocytes were made according to standard techniques with minor modifications repeatedly used in our laboratory [75,76,77]. The chromosome preparations were aged for 12–24 h at +55 °C before their further use in the experimental procedures. 

### 4.4. Fluorescence in Situ Hybridisation (FISH) 

For detection of telomeric regions, fluorescence in situ hybridisation (FISH) with telomeric probes (Telomere PNA FISH/Cy3; DAKO, Glostrup, Denmark) was carried out on the chromosome preparation slides according to the manufacturer’s recommendations, with minor modifications. The slides were incubated in two changes of TBS, pH 7.5 (DAKO, Glostrup, Denmark) for 5 min each at room temperature. Then, the slides were treated with pepsin solution supplemented with 2 M HCl (45 μL of 10% pepsin, 45 μL of 2 M HCl and 50 mL of distilled water) for 5 min at +37 °C and fixed in 3.7% formaldehyde in a TBS buffer for 5 min at room temperature. The preparations were washed twice with TBS for 5 min each, rinsed in distilled water, dehydrated in an ethanol series (70, 80 and 96%) and air dried. Denaturation (10 min at +88 °C) and hybridisation with telomeric probes (Telomere PNA FISH/Cy3; DAKO, Denmark) (18–20 h at +37 °C) were carried out using the ThermoBrite System (Abbott Laboratories, Chicago, IL, USA). After hybridisation, the preparations were washed in Rinse Solution (DAKO Denmark A/S, Denmark) for 1 min at room temperature, washed in Wash Solution (DAKO Denmark A/S, Denmark) for 5 min at +62.5 °C in a shaking bath, rinsed in distilled water and air dried. Then, the preparations were dehydrated in an ethanol series (70, 80 and 96%), air dried and mounted in DAPI-containing VECTASHIELD Antifade (Vector Laboratories, H-1200, Burlingame, CA, USA). The preparations were stored in the dark at +4 °C until the time of microscopic analysis and photoimaging. 

After photoimaging, the detection of the 16p subtelomeric region (the reference region for TL measurements in our study) was carried out using FISH with a TelVysion SpectrumGreen 16p DNA probe (Abbott Laboratories, USA). The coverslips were removed; the preparations were washed with cold water, dehydrated in an ethanol series (70, 80 and 96%) and air dried. Denaturation (10 min at +78 °C) and hybridisation with a 16p subtelomeric probe (Abbott Laboratories, USA) (18–20 h at +37 °C) were carried out using the ThermoBrite System (Abbott Laboratories, USA). After hybridisation, the preparations were washed in 4xSSC supplemented with Tween 20 and in two changes of 4xSSC at +37 °C in a shaking bath. Then, the preparations were rinsed in distilled water, dehydrated in an ethanol series (70, 80 and 96%), air dried and mounted in DAPI-containing VECTASHIELD Antifade (Vector Laboratories, USA). The preparations were stored in the dark at +4 °C until the time of microscopic analysis and photoimaging. 

### 4.5. Immunodetection of 5-Hydroxymethylcytosine (5hmC) and 5-Methylcytosine (5mC) 

The immunodetection of 5hmC and 5mC was carried out on the chromosome preparations from zygotes using primary antibodies against 5hmC (rabbit polyclonal, Active Motif, 39769, Carlsbad, CA, USA) and 5mC (mouse monoclonal, clone 33D3, Millipore, Burlington MA, USA) and secondary goat anti-rabbit Alexa Fluor 488 and goat anti-mouse Alexa Fluor 555 (Life Technologies, A-11008 and A-21424, Carlsbad, CA, USA) antibodies according to the protocol repeatedly used in earlier studies [49,78,79]. The DNA denaturation step routinely used in the immunodetection protocol was omitted, as the denaturation was carried out earlier as part of the FISH procedures. 

### 4.6. Image Acquisition and Evaluation of Telomeric and Subtelomeric FISH Signal Intensity

Fluorescence images of chromosomes after FISH and after immunodetection of 5mC and 5hmC were acquired using the Leica DM 2500 microscope, the Leica DFC345 FX camera and the Leica Application SuiteV.3.8.0 software. Fluorescence images of chromosomes after hybridisation with telomeric DNA probes were acquired using the following acquisition options: exposure time—1.8 s, gain—×3, gamma—3.44. Fluorescence images of chromosomes after hybridisation with the 16p DNA probe were acquired using the following acquisition options: exposure time—2.0 s, gain—×2, gamma—2.0.

The telomeric and subtelomeric FISH signal intensity was evaluated on the digital photoimages using the Image J 1.48v software. Each fluorescent signal area was selected manually with the Freehand Selection tool, and the intensity of the fluorescence in relation to the signal area was measured. 

### 4.7. Statistical Analysis

Statistical analysis was carried out with GraphPad Prism, Version 6.01. The pairwise comparison of TLs between paternal and maternal chromosome sets in zygotes was carried out using the Wilcoxon test. The correlation coefficients were calculated using the non-parametric Spearman test. Non-parametric variables were compared using the Mann–Whitney U test. The variances were compared in IBM SPSS Statistics 23 using Levene’s test. The α-level was set at 0.05.

## Figures and Tables

**Table 1:** Participants’ demographic information, sperm parameters and pronucleus origin.

# Couple	# Individual	Sex	Age	Sperm Parameters			Number of Zygotes Analysed	Triploid Zygote Type by Number of Paternal and Maternal Pronuclei	
				Sperm Concentration, Million/mL	Normal Morphology, %	Progressive Motility, %		2 pat + 1 mat	1 pat + 2 mat
1	1_1	f	23	-	-	-	1	1	
	1_2	m	23	88	4	66			
2	2_1	f	30	-	-	-	2	1	1
	2_2	m	35	59	7	59			
3	3_1	f	31	-	-	-	1	1	
	3_2	m	-	-	-	-			
4	4_1	f	32	-	-	-	1	1	
	4_2	m	34	92	-	67			
5	5_1	f	39	-	-	-	1	1	
	5_2	m	45	136	3	32			
6	6_1	f	32	-	-	-	1	1	
	6_2	m	36	212	2	36			
7	7_1	f	28	-	-	-	1	1	
	7_2	m	29	58	4	17.9			
8	8_1	f	37	-	-	-	2	1	1
	8_2	m	39	56	4	43			
9	9_1	f	31	-	-	-	1	1	
	9_2	m	39	226	12	45.4			
10	10_1	f	38	-	-	-	1	1	
	10_2	m	27	114	0	28			
11	11_1	f	30	-		-	1		1
	11_2	m	33	57	30	41			
12	12_1	f	31	-	-	-	1	1	
	12_2	m	26	169	11	45.8			
13	13_1	f	37	-	-	-	1	1	
	13_2	m	-	230	8.3	57.4			
14	14_1	f	32	-	-	-	1	1	
	14_2	m	33	85	3	62			
15	15_1	f	28	-	-	-	1		1
	15_2	m	28	162	4	52			
16	16_1	f	38	-	-	-	1	1	
	16_2	m	40	42	3	55			
17	17_1	f	32	-	-	-	2	2	
	17_2	m	36	49	12	51			
18	18_1	f	33	-	-	-	1	1	
	18_2	m	-	-	-	-			
19	19_1	f	24	-	-	-	1	1	
	19_2	m	34	120	5	50			
20	20_1	f	32	-	-	-	1	1	
	20_2	m	36	78	0	61			
21	21_1	f	33	-		-	3	3	
	21_2	m	34	117	8	72			
22	22_1	f	31	-		-	2	2	
	22_2	m	31	131	8	52.6			
Total							28	24	4

**Table 2:** Telomere length (TL) characteristics in chromosomes from the maternal and paternal pronuclei of human triploid zygotes.

# Couple	# Individual	Number of Analysed Pronuclei (Gametes)	Mean Relative TL in Pronucleus	Fold Change between Mean Relative TL in Pronucleus and Mean Value across All Pronuclei within the Zygote(s) of the Same Individual	Mean Relative TL across All Pronuclei in the Same Individual	Fold Change between Mean Relative TL across All Pronuclei in the Same Individual and Mean Value across All Individuals
Maternal pronuclei						
1	1_1	1	0.35	-	0.35	2.68
2	2_1	3	0.32	1.02	0.33	2.86
			0.21	1.54		
			0.45	1.37		
3	3_1	1	0.72	-	0.72	1.29
4	4_1	1	0.45	-	0.45	2.09
5	5_1	1	0.81	-	0.81	1.16
6	6_1	1	0.33	-	0.33	2.82
7	7_1	1	0.89	-	0.89	1.05
8	8_1	3	0.44	1.04	0.43	2.19
			0.33	1.29		
			0.51	1.18		
9	9_1	1	0.41	-	0.41	2.27
10	10_1	2	0.88	1.39	1.44	1.54
			2.00			
11	11_1	1	0.48	-	0.48	1.95
12	12_1	1	1.34	-	1.34	1.44
13	13_1	1	0.78	-	0.78	1.42
14	14_1	1	1.32	-	1.32	1.41
15	15_1	2	0.66	1.42	1.13	1.21
			1.60			
16	16_1	1	1.65	-	1.65	1.77
17	17_1	2	0.50	1.52	1.05	1.12
			1.59			
18	18_1	1	0.70	-	0.70	1.33
19	19_1	1	2.73	-	2.73	2.93
20	20_1	1	1.29	-	1.29	1.39
21	21_1	3	0.27	2.07	0.56	1.68
			0.87	1.05		
			0.53	1.56		
22	22_1	2	0.62	1.22	0.79	1.18
			0.96			
Total		32	Mean relative TL across all individuals		0.91	
Paternal pronuclei						
1	1_2	2	0.48	1.04	0.50	3.11
			0.52			
2	2_2	3	0.60	1.31	0.45	3.42
			0.42	1.08		
			0.35	1.32		
3	3_2	2	2.21	1.52	1.45	1.08
			0.69			
4	4_2	2	0.50	1.04	0.48	3.27
			0.46			
5	5_2	2	0.82	1.08	0.89	1.76
			0.96			
6	6_2	2	1.82	1.54	1.18	1.32
			0.54			
7	7_2	2	1.33	1.27	1.05	1.49
			0.77			
8	8_2	3	0.49	1.78	0.87	1.79
			1.13	1.30		
			1.00	1.14		
9	9_2	2	1.03	1.21	1.30	1.20
			1.57			
10	10_2	2	2.86	1.29	1.31	1.19
			1.57			
11	11_2	1	1.31	-	2.22	1.42
12	12_2	2	2.61	1.09	2.86	1.83
			3.10			
13	13_2	2	0.88	1.45	1.58	1.02
			2.29			
14	14_2	2	2.04	1.29	2.87	1.84
			3.69			
15	15_2	1	3.83	-	3.83	2.46
16	16_2	2	3.26	1.00	3.25	2.08
			3.24			
17	17_2	4	0.87	1.54	0.95	1.64
			0.84	1.09		
			1.46	1.13		
			0.63	1.51		
18	18_2	2	0.78	1.07	0.84	1.85
			0.90			
19	19_2	2	1.62	1.11	1.82	1.17
			2.03			
20	20_2	2	2.59	1.11	2.34	1.50
			2.08			
21	21_2	6	0.39	1.23	0.85	1.83
			1.04	2.15		
			0.88	1.04		
			0.96	1.13		
			0.71	1.19		
			1.11	1.31		
22	22_2	4	0.63	1.16	1.41	1.10
			1.21	2.24		
			0.82	1.73		
			2.99	2.12		
Total		52	Mean relative TL across all individuals		1.56	
